# Molecular Detection of Viral and Bacterial Pathogens in Red Foxes (*Vulpes vulpes*) from Italy

**DOI:** 10.3390/ani14131969

**Published:** 2024-07-03

**Authors:** Martina Magliocca, Roberta Taddei, Lorenza Urbani, Cristina Bertasio, Veronica Facile, Laura Gallina, Maria Sampieri, Gianluca Rugna, Silva Rubini, Giulia Maioli, Alessia Terrusi, Mara Battilani, Andrea Balboni

**Affiliations:** 1 Department of Veterinary Medical Sciences, Alma Mater Studiorum-University of Bologna, 40064 Ozzano Emilia, Bologna, Italy; martina.magliocca2@unibo.it (M.M.); lorenza.urbani2@unibo.it (L.U.); veronica.facile2@unibo.it (V.F.); laura.gallina@unibo.it (L.G.); alessia.terrusi2@unibo.it (A.T.); mara.battilani@unibo.it (M.B.); 2 Istituto Zooprofilattico Sperimentale della Lombardia e dell’Emilia Romagna (IZSLER) “Bruno Ubertini”, Sede Territoriale di Bologna, 40127 Bologna, Italy; roberta.taddei@izsler.it (R.T.); maria.sampieri@izsler.it (M.S.); giulia.maioli@izsler.it (G.M.); 3 Italian Reference Centre for Animal Leptospirosis, Istituto Zooprofilattico Sperimentale della Lombardia e dell’Emilia Romagna (IZSLER) “Bruno Ubertini”, Sede Territoriale di Brescia, 25124 Brescia, Italy; cristina.bertasio@izsler.it; 4 Istituto Zooprofilattico Sperimentale della Lombardia e dell’Emilia Romagna (IZSLER) “Bruno Ubertini”, Sede Territoriale di Modena, 41122 Modena, Italy; gianluca.rugna@izsler.it; 5 Istituto Zooprofilattico Sperimentale della Lombardia e dell’Emilia Romagna (IZSLER) “Bruno Ubertini”, Sede Territoriale di Ferrara, 44124 Ferrara, Italy; silva.rubini@izsler.it

**Keywords:** canine adenovirus, canine distemper virus, canine parvovirus type 2, circovirus canine, feline panleukopenia virus, *Leptospira*, protoparvovirus carnivoran 1, wildlife

## Abstract

**Simple Summary:**

Wild animals play an important role in the transmission and maintenance of infectious diseases. In this study, molecular assays were used to detect and genetically characterise Protoparvovirus carnivoran 1 (PPVC-1), Canine adenovirus type 1 and 2 (CAdV-1 and CAdV-2), Circovirus canine (CanineCV), Canine distemper virus (CDV), and *Leptospira* spp. in different matrices collected from 126 red foxes (*Vulpes vulpes*) in Italy from 2022 to 2023. A total of 39 of 126 (30.9%; 95%CI: 23.5–39.5) animals were infected with at least one pathogen. A red fox was coinfected with feline panleukopenia virus (FPV) and canine parvovirus type 2b (CPV-2b) showing quasispecies dynamics. Unique genetic characteristics were identified in CanineCV. The first detection of *L. interrogans* ST198 serogroup Australis in red foxes was also reported. The genetic analysis of the identified PPVC-1, CAdV-1, and *Leptospira* spp. support a wild-to-domestic animals (or vice versa) transmission, while the genetic characteristics of the identified CanineCV confirm a host predilection or limited interspecies transmission of this virus. Further studies are necessary to understand the role of red foxes in the maintenance of these pathogens not only in the wild but also in urban and peri-urban environments.

**Abstract:**

Animals, including wildlife, are part of One-Health concept since many infectious diseases can affect both humans and animals. In this study, 126 red foxes (*Vulpes vulpes*) from Northern Italy in 2022–2023 were tested by molecular assays for Protoparvovirus carnivoran 1 (PPVC-1), Canine adenovirus type 1 and 2 (CAdV-1 and CAdV-2), Circovirus canine (CanineCV), Canine distemper virus (CDV), and *Leptospira* spp. A total of 39 of 126 (30.9%) red foxes were infected with at least one pathogen and five of these were coinfected: 20/126 (15.9%) red foxes tested positive for PPVC-1, 3/126 (2.4%) for CAdV, 20/126 (15.9%) for CanineCV, and 2/126 (1.6%) for *Leptospira* spp. DNA. No foxes tested positive for CDV RNA. The pathogens identified were genetically analysed. New findings were reported such as a fox with multiple feline panleukopenia virus (FPV) and canine parvovirus type 2b (CPV-2b) infection associated with quasispecies dynamics, typical genetic characteristics of the identified CanineCV, and the first detection in red foxes of *Leptospira* ST198 related to *L. interrogans* serogroup Australis. Further studies are necessary to investigate the transmission between domestic animals and wildlife and to understand the role of red foxes in the maintenance of these pathogens not only in the wild but also in urban and peri-urban environments.

## 1. Introduction

Animals, including wildlife, are part of the One-Health concept since many infectious diseases can affect both humans and animals [1]. On the other hand, domestic animals may also be responsible for introducing infectious agents into wildlife, threatening the potential extinction of some animal species [2,3]. The factors that mostly influence the diffusion of infectious diseases in European wild animals are globalisation and global warming. Because natural habitats in decline no longer constitute a suitable environment, many wild animal species tend to move closer to anthropised areas, colonising urban and peri-urban ones [1,4,5,6,7]. The red fox (*Vulpes vulpes*) is a wild carnivore capable of adapting to different environments that can be found in urban, suburban, and rural areas. These behavioural characteristics determine a crucial role in the epidemiology of infectious diseases [8]. However, even red foxes living in exclusively wild environments could be infected by infectious diseases of domestic animals and could represent an important sentinel species [9]. If viral and bacterial pathogens such as Protoparvovirus carnivoran 1 (PPVC-1), Canine mastadenovirus (CAdV), Circovirus canine (CanineCV), Morbillivirus canis or Canine distemper virus (CDV), and *Leptospira* spp. were to become established in red foxes, it could cause severe diseases affecting the conservation of the species and potentially serve as a source of infection for domestic animals or humans.

The PPVC-1, of the *Parvoviridae* family [10], includes two important viral pathogens responsible for gastroenteritis and immunosuppression in domestic and wild carnivores: feline panleukopenia virus (FPV) and canine parvovirus type 2 (CPV-2) [11]. In Europe, the presence of FPV has been documented in several wild animals such as red foxes [12,13], badgers (*Meles meles*) [13,14,15], and stone martens (*Martes foina*) [14], through serology or molecular methods. Similarly, the presence of CPV-2 has been documented in a variety of free-ranging carnivores in Europe, from canids, like foxes and wolves (*Canis lupus*) [12,14,16,17,18,19], to mustelids [13,14,15,20] and ursids like brown bears (*Ursus arctos*) [3]. Transmission between wild and domestic carnivores is supported by many molecular studies that showed the sharing of identical or closely related parvoviruses between these animal species [21,22].

CAdV, of the *Adenoviridae* family [10], includes canine adenovirus type 1 (CAdV-1) and canine adenovirus type 2 (CAdV-2), responsible for infectious canine hepatitis (ICH) and upper respiratory tract infection in dogs [23], respectively. CAdV-1 infection has been reported in several animals belonging to the *Canidae* [24,25,26], *Mustelidae* [27,28], and *Ursidae* [29] families. In wild carnivores, CAdV-1 infection was mainly associated with encephalitis [26,30], although most of the cases reported in the literature suggest a subclinical infection [31,32]. Differently, the exposure of wild carnivores to CAdV-2 has been reported, but its epidemiological role and pathogenicity are not yet clear [33,34].

CanineCV, of the *Circoviridae* family [10], was discovered for the first time in 2011 in serum samples from several dogs [35] and successively reported in wild animals, including foxes [36], wolves, and badgers [37]. To date, its pathogenic role in domestic and wild carnivores is not clarified, but its detection was associated with gastrointestinal and neurological alterations [38,39].

CDV, of the *Paramyxoviridae* family [10], has worldwide distribution and high pathogenic potential [40]. Its detection is associated with a variety of systemic and neurological clinical signs [41,42]. In Europe, the presence of CDV has been documented within the *Canidae* family, including domestic dogs [43] and free-ranging canids, like foxes and wolves [19,44,45,46], and also within the families *Mustelidae* [42,46,47,48,49], *Procyonidae* [46], and *Felidae* [50].

Leptospirosis is a worldwide zoonosis affecting numerous wild and domestic mammalian species [51], caused by pathogenic and highly motile spirochete of the genus *Leptospira*, of the *Leptospiraceae* family [52]. Leptospires are maintained in nature by several subclinical wild and domestic reservoir hosts that serve as exposure sources to wildlife, livestock, domestic animals, and humans [53,54,55,56], and they are transmitted by direct or indirect contact with material, especially soil or water, that has been contaminated with urine from an infected animal [57]. Interestingly, leptospirosis detection has been documented in accidental hosts such as domestic dogs [55,58] and wild animals [59,60], where the clinical signs could be severe and sometimes fatal. 

The aim of this study was to investigate the frequency of infection with viral and bacterial infectious agents causing severe diseases in red foxes in Italy that can be transmitted to domestic animals or humans. For this purpose, a molecular detection of PPVC-1, CAdV, CanineCV, CDV, and *Leptospira* spp. was carried out from internal organs of red foxes in Northern Italy (Emilia Romagna region) in 2022–2023. Genetic characterisation of the identified pathogens was carried out.

## 2. Materials and Methods

### 2.1. Study Design and Sampling

To evaluate the frequency of PPVC-1, CAdV, CanineCV, CDV, and *Leptospira* spp. infection in red foxes, animals that died in Northern Italy for reasons unrelated to the current study were sampled and tested. All the red foxes culled during the regular hunting season or found dead in the environment or dead in wildlife rescue and rehabilitation centres in the Emilia Romagna region (Bologna, Modena, and Ferrara provinces) from January 2022 to March 2023 were included in the study. Animals were collected as part of the wildlife health surveillance program set up in the Emilia Romagna region [61]. No criteria of exclusion were adopted. Each fox was subjected to post-mortem examination at the Istituto Zooprofilattico Sperimentale della Lombardia e dell’Emilia Romagna (IZSLER, Italy) where the intestine, spleen, kidney, and liver were sampled based on availability (the liver was sampled when the spleen was not available) using sterile instruments. Biological samples were stored at −20 °C until further processing. Signalment data (geographical origin, data of sampling, sex, and age) and gross findings were recorded for each animal. Animals were classified as young (<1 year old) or adult (≥1 year old) based on body size and weight. The presence of PPVC-1, CAdV, CanineCV, CDV, and *Leptospira* spp. nucleic acids was detected using quantitative molecular assays. The complete genome or informative genes of the identified pathogens were amplified, sequenced, and analysed.

### 2.2. Detection of Protoparvovirus carnivoran 1, Canine mastadenovirus, Circovirus canine, Canine Distemper Virus, and Leptospira spp. Nucleic Acids

The detection of the nucleic acid of the pathogens investigated was carried out from different tissue samples: PPVC-1 and CanineCV DNA from spleen, liver, and intestine samples; CAdV DNA from spleen, liver, intestine, and kidney samples; CDV RNA from spleen or liver samples; and *Leptospira* spp. DNA from kidney samples.

DNA extraction from all the sampled organs was performed using the NucleoSpin Tissue kit (Macherey-Nagel, Düren, Germany) according to the manufacturer’s instructions. The extracted DNA was eluted in 100 µL of elution buffer and stored at −20 °C until analysis. RNA extraction from spleen and liver samples was performed using the RNeasy kit (Qiagen, Hilden, Germany) according to the manufacturer’s instructions. The extracted RNA was eluted in 40 µL of RNase-free water and stored at −80 °C until analysis.

The detection of PPVC-1 (FPV and CPV-2), CAdV (CAdV-1 and CAdV-2), CanineCV, and *Leptospira* spp. DNA was carried out with four specific SYBR Green real-time PCR (qPCR) assays, each performed using the PowerUp SYBR Green Master Mix (Thermo Fisher Scientific, Life Technologies, Carlsbad, CA, USA), as described in previous studies and in the Supplementary Materials [18,62]. The detection of CDV RNA was carried out with a specific SYBR Green reverse transcriptase qPCR (RT-qPCR) assay, performed using the Power SYBR Green RNA-to-C_T_ 1-Step kit (Life Technologies, Carlsbad, CA, USA), as reported by previous studies [63] and in the Supplementary Materials. Each reaction was carried out using the StepOnePlus Real-Time PCR System (Thermo Fisher Scientific, Life Technologies, Carlsbad, CA, USA). The melting experiment was performed after the last extension step by a continuous increment from 55 °C to 98 °C and the specific melting temperatures were about 77 °C for FPV and CPV-2, 73 °C for CAdV-1, 80 °C for CAdV-2, 93 °C for CanineCV, 78 °C for CDV, and 82 °C for *Leptospira* spp. Nucleic acid copy number determination was carried out by using the standard curve method, as reported by Balboni and colleagues [18], with a limit of detection (LOD) of the reactions of 1 copy/μL for FPV, CPV-2, CAdV-1, CAdV-2, CDV, and *Leptospira* spp., and of 5 copies/μL for CanineCV. In each run, samples and standards were repeated in duplicate and a no template control (ultrapure water) and a negative extraction control were analysed simultaneously.

### 2.3. Sequencing and Analysis of the Viruses Identified

For viruses identified in qPCRs, either the complete genome or the informative genes were amplified, sequenced, and analysed. Each end-point PCR assay was performed using the Phusion Hot Start II DNA Polymerase (Thermo Fisher Scientific, Life Technologies, Carlsbad, CA, USA), containing a high-fidelity DNA polymerase, according to the manufacturer’s instruction. A positive control, consisting of a laboratory positive sample, and a no template control underwent analysis simultaneously (Supplementary Materials).

For the identified PPVC-1, CAdV, and CanineCV, a fragment of the VP2 gene, the complete hexon and fibre genes, and the complete viral genome were amplified [18] and sequenced by the Sanger method (BioFab Research, Rome, Italy), respectively.

Nucleotide sequences obtained from the PCR product of one identified PPVC-1 (lab ID: 829/2022-intestine) showed an unusually high number of ambiguities, suggesting a mixed viral population. Therefore, the amplification product was cloned into the pCR 4/TOPO vector using the pCR 4-TOPO TA kit (Life Technologies, Carlsbad, CA, USA) and transformed into *Escherichia coli* DH5α-competent cells according to the manufacturer’s protocol. The recombinant clones obtained were sequenced after plasmid purification with the PureLink Quick Plasmid Miniprep (Life Technologies, Carlsbad, CA, USA).

The obtained sequences were assembled, analysed with the BLAST web interface (https://blast.ncbi.nlm.nih.gov/Blast.cgi, accessed on 11 December 2023), aligned with reference sequences from the GenBank database (https://www.ncbi.nlm.nih.gov/genbank/, accessed on 11 December 2023) using the ClustalW method implemented in BioEdit software version 7.2.5 (Tom Hall, Ibis Biosciences, Carlsbad, CA, USA), and translated into amino acid sequences.

Phylogeny was carried out on nucleotide sequences obtained in this study and reference sequences obtained in the GenBank database (https://www.ncbi.nlm.nih.gov/nucleotide/, accessed on 25 January 2024) using MEGA 11 software, version 11.0.10 [64]. Subsequently, a Neighbour-Joining phylogenetic tree of the partial VP2 gene of PPVC-1 was constructed using the Tamura 3-parameter model with gamma distribution. Additionally, a Maximum Likelihood phylogenetic tree of the multiple gene sequences (concatenated hexon and fibre gene sequences) of CAdV was constructed using the Hasegawa–Kishino–Yano model with gamma distribution and invariable sites. A Maximum Likelihood phylogenetic tree of the complete genome of CanineCV was constructed using the General Time Reversible model with gamma distribution and invariable sites. One thousand replicates of bootstrap analysis were performed to evaluate the robustness of the phylogenetic trees.

### 2.4. Genotyping by Multi-Locus Sequence Typing of the Leptospira spp. Identified

The identified *Leptospira* spp. were genotyped using the multi-locus sequence typing (MLST) approach, adopting a scheme based on seven housekeeping genes [65]: UDP-N-acetylglucosamine pyrophosphorylase (glmU), NAD(P)(+) transhydrogenase alpha subunit (pntA), 2-oxoglutarate dehydrogenase E1 component (sucA), triosephosphate isomerase (tpiA), 1-phosphofructokinase (pfkB), rod shape-determining protein rodA (mreA), and acyl-CoA transferase/carnitine dehydratase (caiB). A protocol previously developed for direct application on DNA extracted from biological samples was adopted [66]. Nucleotide sequences of each of the seven genes were trimmed and analysed with the Bionumerics Software (version 7.6; Applied-Maths, Sint Maartens-Latem, Belgium) and sequence types (STs) were assigned through the MLST database (https://pubmlst.org/organisms/leptospira-spp, accessed on 12 March 2024). 

A phylogenetic analysis was conducted on the concatemers of the seven MLST genes in MEGA X software version 10.1.8 [67], using the Maximum Likelihood method and Kimura 2-parameter model with a bootstrap analysis based on 1000 replicates. The phylogeny was inferred with some reference sequences downloaded from BIGSdb [68] related to the most common STs found in Italy.

### 2.5. Statistical Analysis

Data were evaluated using standard descriptive statistics and were analysed using the Fisher’s exact test or Pearson’s Chi-squared test. Not available data were excluded from statistical analysis. Statistical significance was set at *p* < 0.05. Statistical analysis was carried out using the MedCalc Statistical Software version 16.8.4 (MedCalc Software bvba).

## 3. Results

### 3.1. Study Population and Sampling

During the study period, 126 red foxes were included (Table 1): 62/126 (49.3%) were male and 56/126 (44.4%) were female, while for the 8/126 (6.3%) remaining animals, this information was not available. Regarding age, 28/126 (22.2%) red foxes were classified as juveniles, 57/126 (45.2%) as adults, and for 41/126 (32.6%), the data were not available. A total of 55 out of 126 (43.6%) red foxes were from the province of Bologna, 66/126 (52.4%) from the province of Modena, and 5/126 (4%) from the province of Ferrara. Carcass examination revealed macroscopic lesions compatible with culling in 79/126 (62.7%) red foxes and with trauma in 30/126 (23.8%), most likely due to motor vehicle collision. In the remaining 17/126 (13.5%) animals, macroscopic findings were mostly lymphadenomegaly, hepatomegaly, splenomegaly, mange, and neurological, gastrointestinal, or renal lesions. Due to the state of conservation of the carcasses, frequently subjected to autolysis of tissues, histological examinations were not carried out. An intestine sample was collected from all the 126 red foxes included in the study; spleen and kidney samples were available for 123/126 and 117/126 red foxes, respectively; and the liver was collected from the 3/126 animals from which the spleen was not collected.

### 3.2. Detection of Viral and Bacterial Infectious Agents

A total of 39 out of 126 (30.9%; 95% confidence interval CI: 23.5–39.5) red foxes tested positive for at least one of the pathogens screened: 34 were positive to one pathogen only and 5 were coinfected. Regarding the coinfected animals, four were positive for two viruses (PPVC-1 and CanineCV) and one was positive for three viruses (PPVC-1, CAdV-2, and CanineCV) (Table S1). Nevertheless, at post-mortem examination, none of these aforementioned animals showed lesions typically attributable to infection with the identified pathogens. No statistical association was found regarding positivity to all pathogens investigated and sex, age, and geographical origin (Table 1).

Specifically, 20 out of 126 (15.9%; 95%CI: 10.5–23.3) red foxes tested positive for PPVC-1 DNA (Table 1): 8 were positive only in the intestine, 6 only in the spleen, and 1 only in liver samples, whereas 5 red foxes were positive in both spleen and intestine samples (Table S1). The overall median quantity of PPVC-1 DNA was 1.2 × 10^1^ copies of target DNA per microliter of extracted DNA (copies/µL) (range 1.9 × 10^0^–4.1 × 10^2^). A total of 3 out of 126 (2.4%; 95%CI: 0.8–6.7) red foxes tested positive for CAdV DNA (Table 1): 2 were positive for CAdV-1 only in the spleen sample (lab IDs: 1784/2022 and 1798/2022) and 1 was positive for CAdV-2 only in the intestine sample (lab ID: 1422/2022) (Table S1). All foxes tested negative for CAdV DNA in the kidney sample. The overall median quantity of CAdV DNA was 1.2 × 10^2^ copies/µL (range 4.4 × 10^1^–5.2 × 10^2^). A total of 20 out of 126 (15.9%; 95%CI: 10.5–23.3) red foxes tested positive for CanineCV DNA (Table 1): 1 was positive only in the intestine and 4 only in spleen samples, whereas 15 were positive in both the spleen and intestine samples (Table S1). The overall median quantity of CanineCV DNA was 3.1 × 10^2^ copies/ µL (range 6.4 × 10^0^–1.3 × 10^7^). CDV RNA was never detected in spleen or liver samples (Table 1). A total of 2 out of 126 (1.6%; 95%CI: 0.4–5.6) red foxes were positive for *Leptospira* spp. DNA in kidney samples (Table 1 and Table S1), with an overall median quantity of target DNA of 5.6 × 10^1^ copies/µL (range 2.4 × 10^1^–8.9 × 10^1^).

### 3.3. Genetic Analysis of the Pathogens Identified

**Figure 1:**
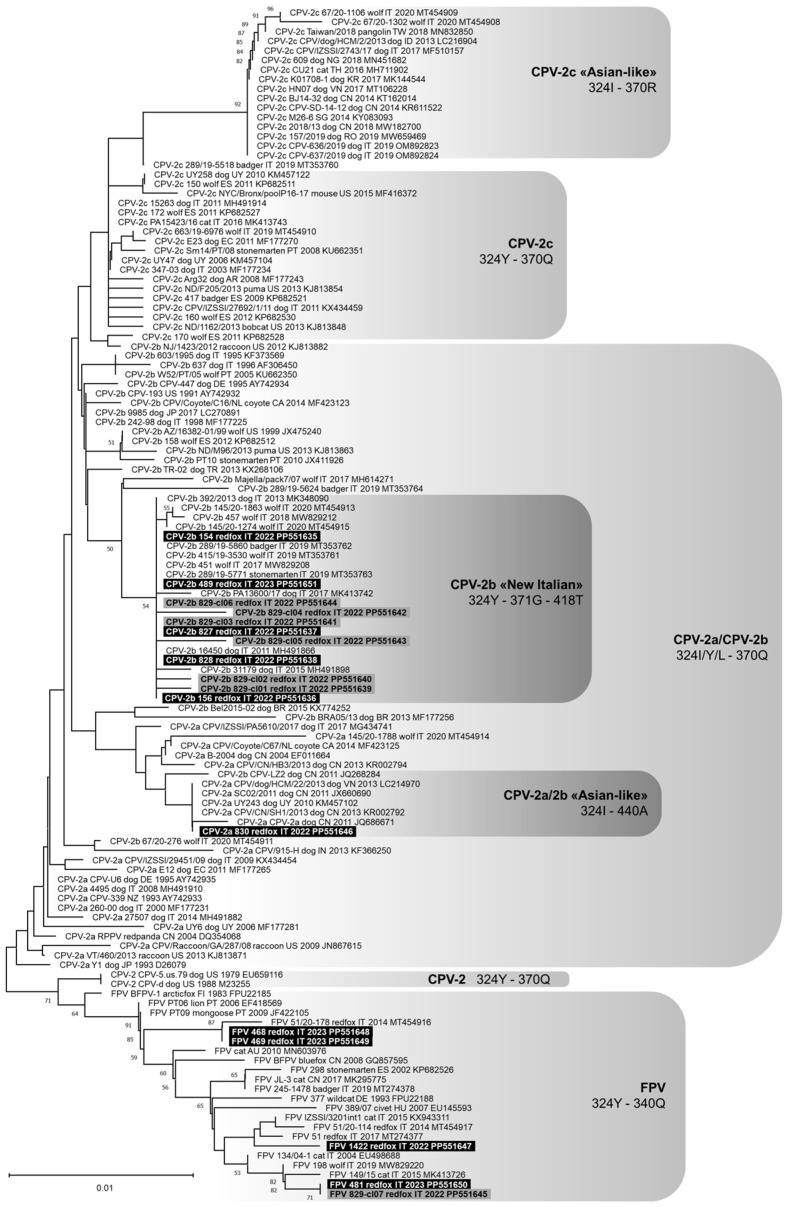
Phylogenetic tree constructed on the partial VP2 nucleotide sequences of Protoparvovirus carnivoran 1 (PPVC-1) obtained in this study and reference strains in the GenBank database, using the Neighbour-Joining method and Tamura 3-parameter model with gamma distribution. One thousand replicates of bootstrap analysis were performed to evaluate the robustness of the phylogenetic trees and bootstrap values ≥ 50% are indicated. Highlighted in black: sequences of canine parvovirus type 2 (CPV-2) and feline panleukopenia virus (FPV) generated in this study. Highlighted in grey: sequences of recombinant clones obtained from red fox 829/2022 in this study. To the right of the figure, the groups evidenced in this study are indicated.

A partial PPVC-1 VP2 gene nucleotide sequence of 532 nts in length (from nt 3669 to 4200 of FPV reference strain CU-4, GenBank ID: M38246) was obtained from 11 foxes. Analysis of the deduced amino acid residues at critical positions allowed us to identify the following viruses: one CPV type 2a (CPV-2a, lab ID: 830/2022), five CPV type 2b (CPV-2b, lab IDs: 154/2022, 156/2022, 827/2022, 828/2022, and 489/2023) with complete nucleotide identity between them, and four FPV-like (lab IDs: 1422/2022, 468/2023, 469/2023, and 481/2023). The nucleotide sequences obtained for FPV 468/2023 and 469/2023 were identical. For 1 out of the 11 PPVC-1 strains (lab IDs: 829/2022), the partial VP2 gene amplicon was cloned and the nucleotide sequence was obtained for seven recombinant clones: six CPV-2b (lab IDs: 829/2022-cl01 to 829/2022-cl06) and one FPV (lab IDs: 829/2022-cl07). The nucleotide sequences of CPV-2b 829/2022-cl03 and cl06 were identical to one another and with the CPV-2b detected in the other red foxes, and FPV 829/2022-cl07 was identical to the FPV identified in red fox 481/2023. The putative VP2 amino acid sequence of CPV-2a 830/2022 had the two distinctive amino acid residues 324-isoleucine (Ile) and 440-alanine (Ala). The putative VP2 amino acid sequence of all the CPV-2b strains identified in this study displayed the two distinctive amino acid residues 371-glicine (Gly) and 418-threonine (Thr) and were identical to each other, with the exception of the 829/2022-cl02 that had the substitution of tryptophan (Trp) to arginine (Arg) in position 414. Phylogenetic analyses showed that the CPV-2a identified in this study was closely related to some CPV-2a strains known as “Asian-like”, which share a typical amino acid composition of the VP2 protein (Figure 1). All the CPV-2b strains identified in this study clustered with viruses detected in dogs and wild carnivores from Italy in the last decade, known as “New Italian CPV-2b”. The FPV-like viruses detected were related to FPV reported in cats and wild carnivores from Europe.

**Figure 2:**
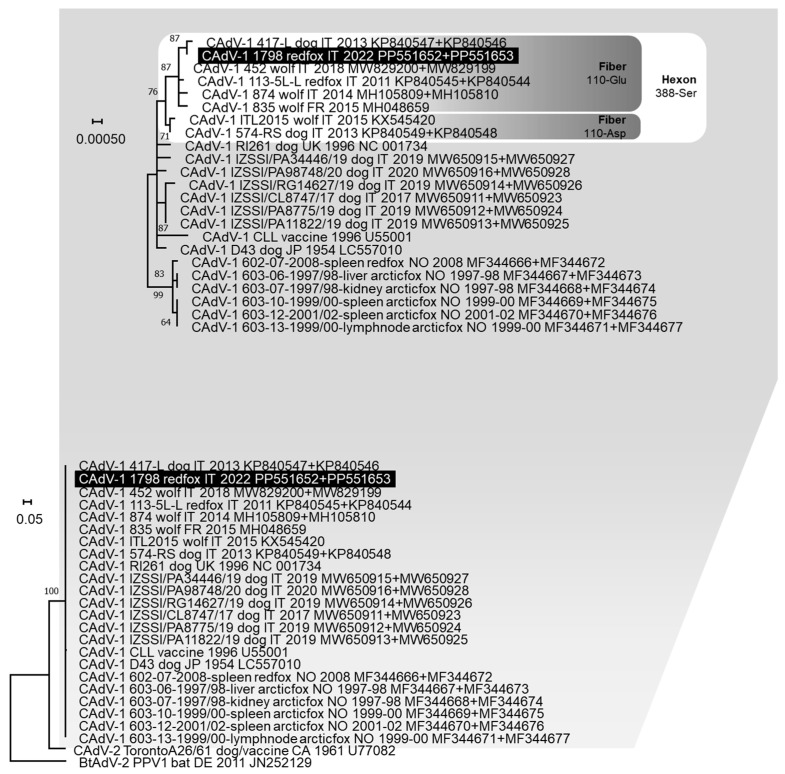
Phylogenetic tree constructed with the multiple gene approach (concatenated nucleotide sequences of the hexon and fibre genes) of Canine mastadenovirus (CAdV) obtained in this study and reference strains in the GenBank database, using the Maximum Likelihood method and Hasegawa–Kishino–Yano (HKY) model with gamma distribution and invariable sites. One thousand replicates of bootstrap analysis were performed to evaluate the robustness of the phylogenetic trees and bootstrap values ≥ 60% are indicated. On the top of the figure, a portion of the obtained tree is enlarged to better visualise the phylogenetic relationships existing between the CAdV-1 nucleotide sequences and the bootstrap values. For some viruses, two GenBank accession numbers are reported (the hexon and fibre genes sequences, respectively). Highlighted in black: nucleotide sequence generated in this study. The amino acid residues in position 388 for the deduced hexon protein and in position 110 for the deduced fibre protein are reported.

Given the low amount of CAdV DNA detected, only incomplete hexon and fibre gene nucleotide sequences of 1600 nts (from nt 676 to 2275 of CAdV-1 reference strain 113-5L, GenBank ID: KP840545) and 1549 nts (from nt 84 to 1632 of CAdV-1 reference strain 113-5L, GenBank ID: KP840544) in length, respectively, were obtained for one CAdV-1 strain (lab ID: 1798/2022). No end-point PCR products were obtained from the other two CAdV strains detected by qPCR. In the phylogenetic tree constructed from the concatenated nucleotide sequences of hexon and fibre genes, CAdV-1 1798/2022 clustered with other CAdV-1 strains identified in domestic and wild canids from Italy and France, characterised by the distinctive residue 388-serine (Ser) in the deduced hexon protein and residue 110-glutamate (Glu) in the deduced fibre protein (Figure 2). 

**Figure 3:**
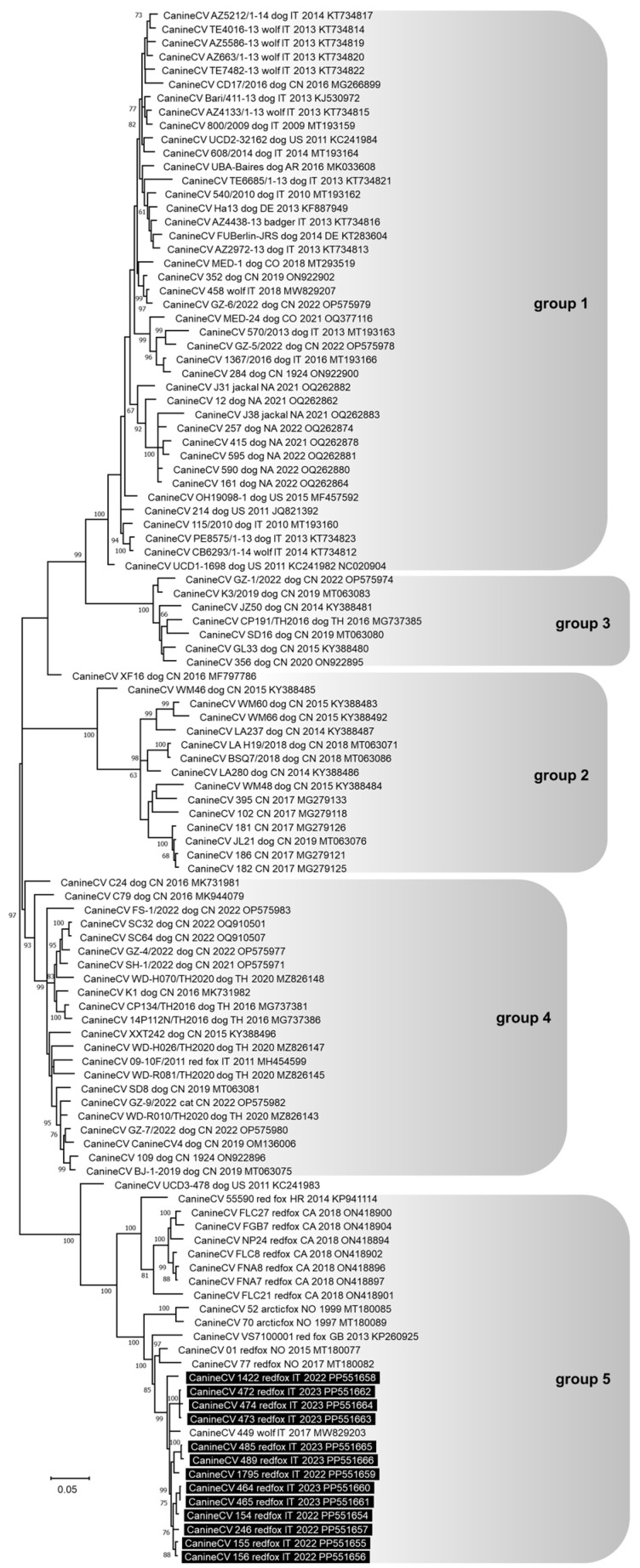
Phylogenetic tree constructed on the complete genome nucleotide sequences of Circovirus canine (CanineCV) obtained in this study and reference strains in the GenBank database, using the Maximum Likelihood method and General Time Reversible (GTR) model with gamma distribution and invariable sites. One thousand replicates of bootstrap analysis were performed to evaluate the robustness of the phylogenetic trees and bootstrap values ≥ 60% are indicated. Highlighted in black: sequences of CanineCV generated in this study. To the right of the figure, the groups evidenced in this study are indicated and correspond to the clusters proposed by Niu et al. [69] and Urbani et al. [36].

The complete genome of 13 CanineCV was sequenced. For nine viruses (lab IDs: 154/2022, 155/2022, 156/2022, 1422/2022, 464/2023, 465/2023, 472/2023, 473/2023, and 474/2023), the genome length was 2063 nts, while for the other four (lab IDs: 246/2022, 1795/2022, 485/2023, and 489/2023), it was 2062 nts. In the latter four CanineCV genomes, the nucleotide deletion involved a guanine in position 959 of reference strain 214, GenBank ID: JQ821392. This deletion was in the non-coding 3’ IR and did not modify the structure of the ORF1 and ORF2. All the CanineCV strains sequenced were different from one another, showing a nucleotide identity of 97–99.9%. In the phylogenetic tree, five groups of CanineCV nucleotide sequences were well distinguishable and all the viruses sequenced in this study clustered in group 5 with viruses identified in foxes and one wolf in Europe (Figure 3). In particular, they were strictly related to a CanineCV strain identified in a wolf in Italy in 2017 (MW829203).

**Figure 4:**
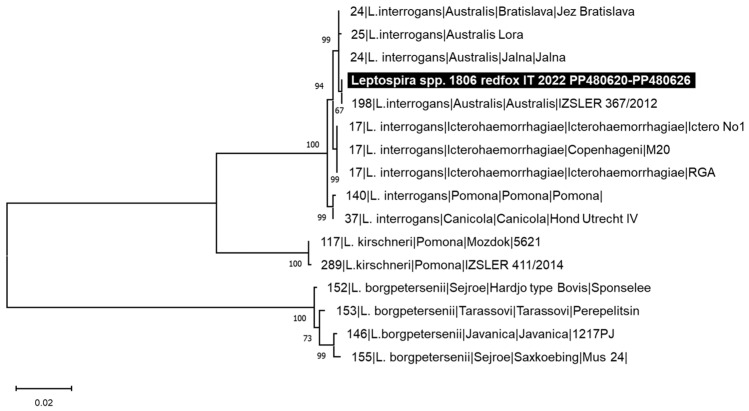
Phylogenetic tree constructed on the concatenated sequences of the seven multi-locus sequence typing (MLST) loci (3111 nts) of *Leptospira* spp. in accordance with the scheme proposed by Boonsilp and colleagues [65]. Phylogeny was conducted using the Maximum Likelihood method and Kimura 2-parameter model. One thousand replicates of bootstrap analysis were performed to evaluate the robustness of the phylogenetic trees and bootstrap values ≥ 60% are indicated. The reference sequences are indicated with ST, species, serogroup, serovar, and strain.

Leptospiral DNA was detected in the kidney sample of two red foxes (lab ID: 1805/2022 and 1806/2022). The identified leptospires were genotyped by MLST analysis and a complete MLST profile was obtained for 1806/2022, while for 1805/2022, a partial profile was defined (Table 2). All two infecting *Leptospira* strains belonged to ST198 that clustered with strain 367/2012, reported as the *L. interrogans* serogroup Australis, serovar Australis (Figure 4). As depicted in Figure 4, ST198 resulted as being very similar to ST24, referred to as the *L. interrogans* serogroup Australis serovars Jalna and Bratislava, having all identical alleles except for the pntA gene.

## 4. Discussion

Wild animals play an important epidemiological role in the maintenance and transmission of infectious diseases, whose causative infectious agents represent a direct threat not only for wild animals but also for domestic animals and humans’ health [1,2,4]. In this study, 39/126 (30.9%) red foxes, that were culled, found dead, or dead in wildlife rescue and rehabilitation centres in Northern Italy (Emilia Romagna region), tested positive to at least one viral or bacterial infectious agent. Post-mortem examination showed no lesions clearly attributable to the pathogens detected, but the advanced state of autolysis, which frequently affected the carcasses, did not allow us to carry out histological examinations and to draw conclusions. 

PPVC-1 and CanineCV were the most frequently detected pathogens, both identified in 20/126 (15.9%) red foxes. In five red foxes, the two viruses were detected, confirming the association already reported for domestic dogs, wolves, and badgers [18,37,70,71]. In dogs, some authors did not observe an aggravation of clinical conditions in animals coinfected by parvovirus and CanineCV [72], while Anderson and colleagues [73] reported an increase in mortality.

Regarding PPVC-1, a lower frequency of positivity in red fox Italian populations was recently reported (2.8% and 5.1%) [13,74]. Conversely, Balboni and colleagues [18] detected a higher frequency of infection in wolves from the same geographical area in Italy (21/23), suggesting that wolves may be more susceptible to infection than foxes. For 11 red foxes that tested positive, a fragment of the PPVC-1 VP2 gene was sequenced, allowing us to identify one CPV-2a virus with a putative amino acid sequence attributable to so-called “Asian-like” viruses never reported before in foxes [75], five CPV-2b viruses with putative amino acid sequences typical of viruses recently circulating in Italy [18], and four FPV-like viruses phylogenetically related to FPV circulating in cats and wild carnivores from Europe. Previous surveys carried out in red foxes in Italy detected only CPV-2 [74] or FPV [13]. These results might suggest a different geographical or temporal distribution of parvoviruses in wildlife. In red fox 829/2022, a multiple infection of FPV and CPV-2b was detected and associated with nucleotide sequence variability of the CPV-2b viral population, resembling the quasispecies distribution already reported for cats [76]. The quasispecies distribution represents a selective advantage favouring viral evolution and the generation of new variants [77], and it could be consistent with the role suggested for wild carnivores in the evolution of carnivore parvoviruses [78,79].

The frequency of CanineCV DNA detection in red foxes obtained in this study was higher than the values reported in Southern Italy by Zaccaria and colleagues (0%) [37] and in Northern Italy by Franzo and colleagues (2–5%) [80]. Contrary to this, an equal or higher prevalence was reported in red foxes from Norway by Urbani and colleagues (16.9%) [36] and from the United Kingdom by Bexton and colleagues (65%) [39], or in wolves from Italy [18,37,81]. The numerous surveys carried out suggest that CanineCV circulates with variable intensity in the different populations of wild canids, with variations probably linked to the investigated host species and to the considered geographical area. Interestingly, the CanineCV genetic group 1 appears mainly widespread in dogs and wolves, whereas CanineCV genetic group 5 appears mainly widespread in foxes, confirming a prevalent species specificity of the different viral genotypes, or a limited transmissibility between different animal species [18,36,37]. Four sequenced CanineCV strains had a nucleotide deletion in the non-coding 3’ intergenic region never reported before and apparently not affecting the functionality of the two main ORFs.

Differently, CAdV and *Leptospira* spp. infections appeared less frequently in red foxes in the investigated area, as they were detected in 3/126 (2.4%) and 2/126 (1.6%) animals, respectively. The frequency of CAdV DNA detection reported in this study is lower than the results of previous surveys conducted in red foxes from neighbouring geographical areas and in wolves from the same geographical area in Italy (9.4% and 17.4%) [18,31]. This finding may reflect a different geographic or host distribution of the virus, as well as variations related to the specific population sampled and the year of investigation. Of the three red foxes that tested positive for CAdV DNA, two were positive for CAdV-1 and one was positive for CAdV-2. Furthermore, in one of the red foxes that tested positive for CAdV-2, PPVC-1 and CanineCV DNA were also detected, highlighting a possible coinfection with three viruses sporadically reported in wolves [18]. The CAdV-1 identified in this study was phylogenetically grouped with all CAdV-1 strains detected in domestic dogs and wild carnivores from Italy and France and showed the residues 388-Ser in the deduced hexon protein and 110-Glu in the deduced fibre protein. These results support the hypothesis that the same viruses circulate in domestic and wild environments [18] and confirm that some amino acid positions may allow us to distinguish viruses circulating in different geographical regions [18].

The red fox, like the domestic dog, is considered an accidental host for *Leptospira* infection and its role was investigated considering that the main food of foxes is represented by rodents, the most important reservoir host for several *Leptospira* serovars [82]. *Leptospira* spp. infection in foxes not only represents a possible source of environmental contamination [83] but may also cause illness and death [84,85]. The majority of the surveys carried out to evaluate the prevalence of *Leptospira* spp. infection in foxes used the microscopic agglutination test (MAT), and in Europe, the predominant serogroups detected were Australis, Bataviae, Grippotyphosa, Hebdomadis, Icteroheamorrhagiae, Javanica, Pomona, and Sejroe [56,59,86,87,88,89,90,91,92,93]. Only few surveys carried out on red foxes adopted molecular-based assays such as qPCR or MLST [93,94,95,96,97]. On one hand, the MAT assay detecting specific antibodies allows us to obtain more complete indications than molecular methods regarding the circulation of leptospires in an animal population, but on the other hand, using a limited antigens panel can lead to an underestimation of the serovars. Molecular methods do not have this limitation, therefore, qPCR and MLST assays allow for the detection of serovars not included in the MAT antigen panel. The frequency of *Leptospira* spp. DNA detection obtained in this study is similar to that reported for red foxes from neighbouring geographical areas by Ebani and colleagues [96]. Furthermore, MLST analysis allowed us to identify *Leptospira* ST198 related to *L. interrogans* serogroup Australis serovar Australis in both red foxes testing positive in this study. The Australis serogroup has been widely reported in dogs in Italy [55,62], and *Leptospira* ST198 was previously reported in a horse [55], in hedgehogs, and in association with severe disease in dogs in Italy [58,98]. To the best of the authors’ knowledge, this represents the first report of *Leptospira* ST198 in red foxes, suggesting that this variant is widespread in the Italian territory, involving different animal species acting as maintenance or accidental hosts, and confirming that the red fox may be a reliable sentinel for epidemiological monitoring [92].

In the present study, none of the red foxes analysed were found positive for CDV RNA. Although it cannot be ruled out that viral RNA lability in the environment may have affected the result obtained, the lack of positive findings is in line with the Italian epidemiological situation where CDV circulates in wild animals in the Alps [45,99] but is detected sporadically on the rest of the territory [49,100,101].

For PPVC-1, CAdV-1, and *Leptospira* spp., the amount of genomic nucleic acid detected was low, not exceeding the order of magnitude of 10^2^. These values are potentially correlated to states of subclinical or even persistent infection, typical of the carrier state or maintenance host, often hypothesised for the red fox [17,74], and compatible with the absence of characteristic lesions detected at post-mortem examination. Differently, in some foxes, the amount of CanineCV DNA detected reached up to 10^7^ copies/uL. Similar quantities were also reported in intestine and spleen samples of wolves [18], suggesting an active infection dynamic for this virus, although not necessarily associated with clinical signs.

The present study has some limitations. Firstly, samples were only collected from dead animals that were often found hours or days after death, and this could have affected the integrity of the nucleic acids, resulting in an underestimation of the pathogens detected (especially CDV which has an RNA genome). Secondly, the study was based exclusively on the use of molecular methods that detect the presence of the genome of the infectious agents but not their vitality. So, positive results may not correspond to the real state of infection, but they are nevertheless indicative of the circulation of pathogens in wildlife. Furthermore, molecular tests give an indication of the presence of the agent at the time of sampling only and the combination of a serological test could have allowed us to obtain more complete information on the circulation of these infectious agents in the red fox population.

## 5. Conclusions

This study reports new data on the presence of PPVC-1, CAdV, CanineCV, CDV, and *Leptospira* spp. in red foxes and underlines the importance of this wild carnivore in the epidemiology of several infectious agents. In particular, new findings are reported here such as a fox with multiple FPV and CPV-2b infection associated with quasispecies dynamics, typical genetic characteristics of the identified CanineCV, and the first detection of *Leptospira* ST198 related to the *L. interrogans* serogroup Australis in red foxes. Furthermore, the genetic analysis of the identified PPVC-1, CAdV-1, and *Leptospira* spp. supports a wild-to-domestic (or vice versa) transmission, and the genetic characteristics of the identified CanineCV confirm a host predilection or limited interspecies transmission of this virus. Further studies are necessary to investigate the transmission between domestic animals and wildlife and to understand the role of red foxes in the maintenance of these pathogens not only in the wild but also in urban and peri-urban environments.

## Figures and Tables

**Table 1:** Red foxes included in this study and tested for Protoparvovirus carnivoran 1, Canine mastadenovirus, Circovirus canine, Canine distemper virus, and *Leptospira* spp. nucleic acids.

	Total	PPVC-1	CAdV	CanineCV	CDV	*Leptospira* spp.	Total of Positive Red Foxes	*p* Value
Number of foxes	126	20/126 (15.9; 10.5–23.3)	3/126 (2.4; 0.8–6.7)	20/126 (15.9; 10.5–23.3)	0/126 (0)	2/126 (1.6; 0.4–5.6)	39/126 (30.9; 23.5–39.5)	
Sex								
Male	62/126 (49.3)	9/62 (14.5; 7.8–25.3)	1/62 (1.6; 0.3–8.6)	8/62 (12.9; 6.7–23.5)	0/62 (0)	2/62 (3.2; 0.9–11)	18/62 (29; 19.2–41.3)	0.56
Female	56/126 (44.4)	10/56 (17.9; 10–29.8)	2/56 (3.6; 0.9–12.1)	12/56 (21.4; 12.7–33.8)	0/56 (0)	0/56 (0)	20/56 (35.7; 24.5–48.8)	
NA	8/126 (6.3)	1/8 (12.5; 2.2–47.1)	0/8 (0)	0/8 (0)	0/8 (0)	0/8 (0)	1/8 (12.5; 2.2–47.1)	
Age								
Young (<1 year old)	28/126 (22.2)	7/28 (25; 12.7–43.4)	0/28 (0)	4/28 (14.3; 5.7–31.5)	0/28 (0)	0/28 (0)	10/28 (35.7; 20.7–54.2)	0.89
Adult (≥1 year old)	57/126 (45.2)	11/57 (19.3; 11.1–31.3)	2/57 (3.5; 0.9–11.9)	13/57 (22.8; 13.8–35.2)	0/57 (0)	0/57 (0)	21/57 (36.8; 25.5–49.8)	
NA	41/126 (32.6)	2/41 (4.9; 1.4–16.1)	1/41 (2.4; 0.4–12.6)	3/41 (7.3; 2.5–19.4)	0/41 (0)	2/41 (4.9; 1.4–16.1)	8/41 (19.5; 10.2–34)	
Geographical origin								
Bologna	55/126 (43.6)	12/55 (21.8; 12.9–34.4)	0/55 (0)	3/55 (5.5; 1.9–14.9)	0/55 (0)	0/55 (0)	15/55 (27.3; 17.3–40.2)	0.17
Modena	66/126 (52.4)	8/66 (12.1; 6.3–22.1)	3/66 (4.5; 1.6–12.5)	17/66 (25.8; 16.8–37.4)	0/66 (0)	2/66 (3; 1.4–16.1)	24/66 (36.4; 25.8–48.4)	
Ferrara	5/126 (4)	0/5 (0)	0/5 (0)	0/5 (0)	0/5 (0)	0/5 (0)	0/5 (0)	

The Fisher’s exact test or Pearson’s Chi-squared test were carried out on the total of positive red foxes. Statistical significance was set at *p* < 0.05. Data are reported as n (%) or n (%; 95% confidence interval). Not available data were excluded from statistical analysis. PPVC-1: Protoparvovirus carnivoran 1; CAdV: Canine mastadenovirus; CanineCV: Circovirus canine; CDV: Canine distemper virus; NA: not available.

**Table 2:** Multi-locus sequence typing results of the *Leptospira* spp. identified.

Red Fox	ST	glmU	pntA	pfkB	caiB	mreA	sucA	tpiA
1805/2022	198	1	66	5	4	ND	2	1
1806/2022	198	1	66	5	4	3	2	1

glmU: UDP-N-acetylglucosamine pyrophosphorylase; pntA: NAD(P)(+) transhydrogenase alpha subunit; sucA: 2-oxoglutarate dehydrogenase E1 component; tpiA: triosephosphate isomerase; pfkB: 1-phosphofructokinase; mreA: rod shape-determining protein rodA; caiB: acyl-CoA transferase/carnitine dehydratase; ST: sequence type; ND: not defined.

## Data Availability

All data generated or analysed during this study are included in this published article and its supplementary information files. The nucleotide sequences generated and analysed during the current study are available in the International Nucleotide Sequence Database Collaboration repository (INSDC, http://www.insdc.org/) with the IDs PP480614–PP480626 and PP551635–PP551666.

## Supplementary Materials

The following supporting information can be downloaded at https://www.mdpi.com/article/10.3390/ani14131969/s1. Supplementary Materials: Details on *Protoparvovirus carnivoran* 1, *Canine mastadenovirus*, *Circovirus canine*, *Canine distemper virus*, and *Leptospira* spp. nucleic acids’ detection and nucleotide sequencing for genetic characterisation. Table S1: Red foxes positive for at least one pathogen and gross findings.

## References

[1] Yon L., Duff J.P., Ågren E.O., Erdélyi K., Ferroglio E., Godfroid J., Hars J., Hestvik G., Horton D., Kuiken T. (2019). Recent changes in infectious diseases in European wildlife. J. Wildl. Dis..

[2] Clifford D.L., Mazet J.A.K., Dubovi E.J., Garcelon D.K., Coonan T.J., Conrad P.A., Munson L. (2006). Pathogen exposure in endangered island fox (*Urocyon littoralis*) populations: Implication for conservation management. Biol. Conserv..

[3] Di Francesco C.E., Gentile L., Di Pirro V., Ladiana L., Tagliabue S., Marsilio F. (2015). Serologic evidence for selected infectious diseases in Marsican brown bears (*Ursus arctos marsicanus*) in Italy (2004–09). J. Wildl. Dis..

[4] Riley S.P.D., Foley J., Chomel B. (2004). Exposure to feline and canine pathogens in bobcats and gray foxes in urban and rural zones of a national park in California. J. Wildl. Dis..

[5] Martin C., Pastoret P.P., Brochier B., Humblet M.F., Saegerman C. (2011). A survey of the transmission of infectious diseases/infections between wild and domestic ungulates in Europe. Vet. Res..

[6] Dellamaria D., Citterio C.V., Capelli G., Paternolli S., Turchetto S., Obber F., Cazzin S., Francione E. Principali Patologie della Fauna: Conoscerle e Riconoscerle, 2014. https://issuu.com/izsvenezie/docs/patologie-fauna-selvatica.

[7] Brandell E.E., Cross P.C., Craft M.E., Smith D.W., Dubovi E.J., Gilbertson M.L.J., Wheeldon T., Stephenson J.A., Barber-Meyen S., Borg B.L. (2021). Patterns and processes of pathogen exposure in gray wolves across North America. Sci. Rep..

[8] Garcês A., Pires I. (2021). Secrets of the astute red fox (*Vulpes vulpes*, Linnaeus, 1758): An inside-ecosystem secret agent serving One Health. Environments.

[9] Pluemer M., Dubai S., Drake D., Crimmins S., Veverka T., Hovanec H., Torkelson M., Mueller M. (2019). Red foxes (*Vulpes vulpes*) and coyotes (*Canis latrans*) in an urban landscape: Prevalence and risk factors for disease. J. Urban Ecol..

[10] International Committee on Taxonomy of Viruse (ICTV). https://ictv.global/taxonomy/.

[11] Sykes J.E. (2023). Infectious Diseases of the Dog and Cat.

[12] Duarte M.D., Henriques A.M., Barros S.C., Fagulha T., Mendonca P., Carvalho P., Monteiro M., Fevereiro M., Basto M.P., Rosalino L.M. (2013). Snapshot of viral infections in wild carnivores reveals ubiquity of parvovirus and susceptibility of egyptian mongoose to feline panleukopenia virus. PLoS ONE.

[13] Ndiana L.A., Lanave G., Desario C., Berjaoui S., Alfano F., Puglia I., Fusco G., Colaianni M.L., Vincifori G., Camarda A. (2020). Circulation of diverse protoparvoviruses in wild carnivores, Italy. Transbound. Emerg. Dis..

[14] Calatayud O., Esperón F., Velarde R., Oleaga Á., Llaneza L., Ribas A., Negre N., de la Torre A., Rodríguez A., Millán J. (2019). Genetic characterization of Carnivore Parvoviruses in Spanish wildlife reveals domestic dog and cat-related sequences. Transbound. Emerg. Dis..

[15] Leopardi S., Milani A., Cocchi M., Bregoli M., Schivo A., Leardini S., Festa F., Pastori A., de Zan G., Gobbo F. (2022). Carnivore protoparvovirus 1 (CPV-2 and FPV) circulating in wild carnivores and in puppies illegally imported into North-Eastern Italy. Viruses.

[16] Battilani M., Scagliarini A., Tisato E., Turilli C., Jacoboni I., Casadio R., Prosperi S. (2001). Analysis of canine parvovirus sequences from wolves and dogs isolated in Italy. J. Gen. Virol..

[17] Sobrino R., Arnal M.C., Luco D.F., Gortazar C. (2008). Prevalence of antibodies against canine distemper virus and canine parvovirus among foxes and wolves from Spain. Vet. Microbiol..

[18] Balboni A., Urbani L., Delogu M., Musto C., Fontana M.C., Merialdi G., Lucifora G., Terrusi A., Dondi F., Battilani M. (2021). Integrated use of molecular techniques to detect and genetically characterise DNA viruses in Italian wolves (*Canis lupus italicus*). Animals.

[19] Ferrara G., Brocherel G., Falorni B., Gori R., Pagnini U., Montagnaro S. (2023). A retrospective serosurvey of selected pathogens in red foxes (*Vulpes vulpes*) in the Tuscany region, Italy. Acta Vet. Scand..

[20] Frölich K., Streich W.J., Fickel J., Jung S., Truyen U., Hentschke J., Dedek J., Prager D., Latz N. (2005). Epizootiologic investigations of parvovirus infections in free-ranging carnivores from Germany. J. Wildl. Dis..

[21] Steinel A., Parrish C.R., Bloom M.E., Truyen U. (2001). Parvovirus infections in wild carnivores. J. Wildl. Dis..

[22] Kurucay H.M., Tamer C., Muftuoglu B., Elhag A.E., Gozel S., Cicek-Yildiz Y., Demirtas S., Ozan E., Albayrak H., Okur-Gumusova S. (2023). First isolation and molecular characterization of canine parvovirus-type 2b (CPV-2b) from red foxes (*Vulpes vulpes*) living in the wild habitat of Turkey. Virol. J..

[23] Decaro N., Martella V., Buonavoglia C. (2008). Canine adenovirus and herpesvirus. Veterinary Clinics of North America. J. Small Anim. Pract..

[24] Pizzurro F., Marcacci M., Zaccaria G., Orsini M., Cito F., Rosamilia A., Di Renzo L., Malatesta D., Di Sabatino D., Lorusso A. (2017). Genome sequence of canine Adenovirus type 1 isolated from a wolf (*Canis lupus*) in southern Italy. Genome Announc..

[25] Oleaga A., Balseiro A., Espi A., Royo L.J. (2022). Wolf (*Canis lupus*) as canine adenovirus type 1 (CAdV-1) sentinel for the endangered cantabrian brown bear (*Ursus arctos arctos*). Transbound. Emerg. Dis..

[26] Silva M.L., Caiaffa M.G., da Costa A.L.M., Teixeira R.H.F., Ervedosa T.B., Machado E.F., Suárez P.E.N., Réssio R.A., Borges C.C., de Jesus I.P. (2023). Canine distemper virus and canine adenovirus type 1 co-infection in a free-living hoary fox (*Lycalopex vetulus*) from Brazil. Braz. J. Microbiol..

[27] Junge E.R., Bauman K., King M., Gompper M.E. (2007). A serologic assessment of exposure to viral pathogens and Leptospira in an urban raccoon (*Procyon lotor*) population inhabiting a large zoological park. J. Zoo Wildl. Med..

[28] Hou J., Xu J., Wang B., Zhang H., Yin B., Li G., Lei F., Cai X., Zhu Y., Wang L. (2023). First identification of canine adenovirus 1 in mink and bioinformatics analysis of its 100 K protein. Front. Microbiol..

[29] García Marín J.F., Royo L.J., Oleaga A., Gayo E., Alarcia O., Pinto D., Martínez I.Z., González P., Balsera R., Marcos J.L. (2018). Canine adenovirus type 1 (CAdV-1) in free-ranging European brown bear (*Ursus arctos arctos*): A threat for Cantabrian population?. Transbound. Emerg. Dis..

[30] Green R.G., Ziegler N.R., Green B.B., Dewey E.T. (1930). Epizootic fox encephalitis. Am. J. Trop. Med. Hyg..

[31] Balboni A., Verin R., Morandi F., Poli A., Prosperi S., Battilani M. (2013). Molecular epidemiology of canine adenovirus type 1 and type 2 in free-ranging red foxes (*Vulpes vulpes*) in Italy. Vet. Microbiol..

[32] Walker D., Fee S.A., Hartley G., Learmount J., O’Hagan M.J.H., Meredith A.L., de C. Bronsvoort B.M., Porphyre T., Sharp C.P., Philbey A.W. (2016). Serological and molecular epidemiology of canine adenovirus type 1 in red foxes (*Vulpes vulpes*) in the United Kingdom. Sci. Rep..

[33] Di Francesco C.E., Smoglica C., Paoletti B., Angelucci S., Innocenti M., Antonucci A., Di Domenico G., Marsilio F. (2019). Detection of selected pathogens in Appennine wolf (*Canis lupus italicus*) by a non-invasive GPS-based telemetry sampling of two packs from Majella National Park, Italy. J. Wildl. Res..

[34] Kim Y.J., Lee S.Y., Kim Y.S., Na E.J., Park J.S., Oem J.K. (2022). Genetic characteristics of canine adenovirus type 2 detected in wild raccoon dogs (*Nyctereutes procyonoides*) in Korea. Vet. Sci..

[35] Kapoor A., Dubovi E.J., Henriquez-Rivera J.A., Lipkin W.I. (2012). Complete genome sequence of the first Canine circovirus. J. Virol..

[36] Urbani L., Tryland M., Ehrich D., Fuglei E., Battilani M., Balboni A. (2021). Ancient origin and genetic segregation of canine circovirus infecting arctic foxes (*Vulpes lagopus*) in Svalbard and red foxes (*Vulpes vulpes*) in Northern Norway. Transbound. Emerg. Dis..

[37] Zaccaria G., Malatesta D., Scipioni G., Di Felice E., Campolo M., Casaccia C., Savini G., Di Sabatino D., Lorusso A. (2016). Circovirus in domestic and wild carnivores: An important opportunistic agent?. Virology.

[38] Decaro N., Martella V., Desario C., Lanave G., Circella E., Cavalli A., Elia G., Camero M., Buonavoglia C. (2014). Genomic characterization of a circovirus associated with fatal hemorrhagic enteritis in dog, Italy. PLoS ONE.

[39] Bexton S., Wiersma L.C., Getu S., Van Run P.R., Verjans G.M.G.M., Schipper D., Schapendonk C.M.E., Bodewes R., Oldroyd L., Haagmans B.L. (2015). Detection of circovirus in foxes with meningoencephalitis, United Kingdom, 2009–2013. Emerg. Infect. Dis..

[40] Origgi F.C., Plattet P., Sattler U., Robert N., Casaubon J., Mavrot F., Pewsner M., Wu N., Giovannini S., Oevermann A. (2012). Emergence of Canine Distemper Virus strains with modified molecular signature and enhanced neuronal tropism leading to high mortality in wild carnivores. Vet. Pathol..

[41] Beineke A., Baumgärtner W., Wohlsein P. (2015). Cross-species transmission of canine distemper virus-an update. One Health.

[42] Zhao J., Shi N., Sun Y., Martella V., Nikolin V., Zhu C., Zhang H., Hu B., Bai X., Yan X. (2015). Pathogenesis of canine distemper virus in experimentally infected raccoon dogs, foxes, and minks. Antivir. Res..

[43] Martella V., Lucente M.S., Cirone F., Lorusso E., Elia G., Camero M., Buonavoglia C. (2007). Canine distemper virus (CDV): From the arctic ecosystem to Italy. Veterinaria.

[44] Di Sabatino D., Lorusso A., Di Francesco C.E., Gentile L., Di Pirro V., Bellacicco A.L., Giovannini A., Di Francesco G., Marruchella G., Marsilio F. (2014). Arctic lineage-Canine Distemper Virus as a cause of death in Appenine wolves (*Canis lupus*) in Italy. PLoS ONE.

[45] Bianco A., Zecchin B., Fusaro A., Schivo A., Ormelli S., Bregoli M., Citterio C.V., Obber F., Dellamaria D., Trevisiol K. (2020). Two waves of canine distemper virus showing different spatio-temporal dynamics in Alpine wildlife (2006–2018). Infect. Genet. Evol..

[46] Kličková E., Černíková L., Dumondin A., Bártová E., Budíková M., Sedlák K. (2022). Canine distemper virus in wild carnivore populations from the Czech Republic (2012–2020): Occurrence, geographical distribution, and phylogenetic analysis. Life.

[47] Monne I., Fusaro A., Valastro V., Citterio C., Dalla Pozza M., Obber F., Trevisiol K., Cova M., De Benedictis P., Bregoli M. (2011). A distinct CDV genotype causing a major epidemic in Alpine wildlife. Vet. Microbiol..

[48] Di Sabatino D., Di Francesco G., Zaccaria G., Malatesta D., Brugnola L., Marcacci M., Portanti O., De Massis F., Savini G., Teodori L. (2016). Lethal distemper in badgers (*Meles meles*) following epidemic in dogs and wolves. Infect. Genet. Evol..

[49] Balboni A., Savini F., Scagliarini A., Berti E., Naldi M., Urbani L., Fontana M.C., Carra E., Gibelli L.R.M., Gobbo F. (2021). Natural distemper infection in stone martens (*Martes foina*): From infection to neutralizing antibodies. Res. Vet. Sci..

[50] Kadam R.G., Karikalan M., Siddappa C.M., Mahendran K., Srivastava G., Rajak K.K., Bhardwaj Y., Varshney R., War Z.A., Singh R. (2022). Molecular and pathological screening of canine distemper virus in Asiatic lions, tigers, leopards, snow leopards, clouded leopards, leopard cats, jungle cats, civet cats, fishing cat, and jaguar of different states, India. Infect. Genet. Evol..

[51] Bharti A.R., Nally J.E., Ricaldi J.N., Matthias M.A., Diaz M.M., Lovett M.A., Levett P.N., Gilman R.H., Willig M.R., Gotuzzo E. (2003). Leptospirosis: A zoonotic disease of global importance. Lancet Infect. Dis..

[52] Cilia G., Bertelloni F., Fratini F. (2020). Leptospira infections in domestic and wild animals. Pathogens.

[53] Tan C.G., Dharmarajan G., Beasley J., Rhodes O., Moore G., Wu C.C., Lin T.L. (2014). Neglected leptospirosis in raccoons (*Procyon lotor*) in Indiana, USA. Vet. Quart..

[54] López M.C., Vila A., Rodón J., Roura X. (2019). Leptospira seroprevalence in owned dogs from Spain. Heliyon.

[55] Bertasio C., Boniotti M.B., Lucchese L., Ceglie L., Bellinati L., Mazzucato M., Furlanello T., D’Incau M., Natale A. (2020). Detection of new Leptospira genotypes infecting symptomatic dogs: Is a new vaccine formulation needed?. Pathogens.

[56] Roquelo C., Kodjo A., Marié J.L., Davoust B. (2021). Serological and molecular survey of Leptospira spp. infections in wild boars and red foxes from Southeastern France. Vet. World.

[57] Ko A.I., Goarant C., Picardeau M. (2009). Leptospira: The dawn of the molecular genetics era for an emerging zoonotic pathogen. Nat. Rev. Microbiol..

[58] Balboni A., Zamagni S., Bertasio C., Boniotti M.B., Troìa R., Battilani M., Dondi F. (2020). Identification of serogroups Australis and icterohaemorrhagiae in two dogs with a severe form of acute Leptospirosis in Italy. Pathogens.

[59] Åkerstedt J., Lillehaug A., Larsen I.L., Eide N.E., Arnemo J.M., Handeland K. (2010). Serosurvey for Canine Distemper Virus, Canine Adenovirus, *Leptospira interrogans* and *Toxoplasma gondii* in free-ranging canids in Scandinavia and Svalbard. J. Wildl. Dis..

[60] Mazzotta E., Bellinati L., Bertasio C., Boniotti M.B., Lucchese L., Ceglie L., Martignago F., Leopardi S., Natale A. (2023). Synanthropic and wild animals as sentinels of zoonotic agents: A study of *Leptospira* genotypes circulating in northeastern Italy. Int. J. Environ. Res. Public Health.

[61] Regione Emilia Romagna Sorveglianza e Monitoraggio della Fauna Selvatica. https://servizissiir.regione.emilia-romagna.it/deliberegiunta/servlet/AdapterHTTP?action_name=ACTIONRICERCADELIBERE&operation=dettaglioByDatiAdozione&ENTE=1&TIPO_ATTO=DL&ANNO_ADOZIONE=2017&NUM_ADOZIONE=1763.

[62] Troìa R., Balboni A., Zamagni S., Frigo S., Magna L., Perissinotto L., Battilani M., Dondi F. (2018). Prospective evaluation of rapid point-of-care tests for the diagnosis of acute leptospirosis in dogs. Vet. J..

[63] Scagliarini A., Dal Pozzo F., Gallina L., Vaccari F., Morganti L. (2007). TaqMan based real time PCR for the quantification of canine distemper virus. Vet. Res. Commun..

[64] Tamura K., Stecher G., Kumar S. (2021). MEGA11: Molecular evolutionary genetics analysis version 11. Mol. Biol. Evol..

[65] Boonsilp S., Thaipadungpanit J., Amornchai P., Wuthiekanun V., Bailey M.S., Holden M.T., Zhang C., Jiang X., Koizumi N., Taylor K. (2013). A single multilocus sequence typing (MLST) scheme for seven pathogenic *Leptospira* species. PLoS Negl. Trop. Dis..

[66] Weiss S., Menezes A., Woods K., Chanthongthip A., Dittrich S., Opoku-Boateng A., Kimuli M., Chalker V. (2016). An extended multilocus sequence typing (MLST) scheme for rapid direct typing of *Leptospira* from clinical samples. PLoS Negl. Trop. Dis..

[67] Kumar S., Stecher G., Li M., Knyaz C., Tamura K. (2018). MEGA X: Molecular Evolutionary Genetics Analysis across computing platforms. Mol. Biol. Evol..

[68] Jolley K.A., Bray J.E., Maiden M.C.J. (2018). Open-access bacterial population genomics: BIGSdb software, the PubMLST.org website and their applications. Wellcome Open Res..

[69] Niu L., Wang Z., Zhao L., Wang Y., Cui X., Shi Y., Chen H., Ge J. (2020). Detection and molecular characterization of canine circovirus circulating in northeastern China during 2014–2016. Arch. Virol..

[70] Thaiwong T., Wise A.G., Maes R.K., Mullaney T., Kiupel M. (2016). Canine circovirus 1 (CaCV-1) and canine parvovirus 2 (CPV-2): Recurrent dual infections in a papillon breeding colony. Vet. Pathol..

[71] Faraji R., Sadeghi M., Mozhgani S.H., Vasinioti V., Ndiana L.A., Desario C., Beikpour F., Decaro N. (2022). Detection of canine circovirus in dogs infected with canine parvovirus. Acta Tropica.

[72] da Rocha Gizzi A.B., Oliveira S.T., Leutenegger C.M., Estrada M., Kozemjakin D.A., Stedile R., Marcondes M., Biondo A.W. (2014). Presence of infectious agents and co-infections in diarrheic dogs determined with a real-time polymerase chain reaction-based panel. BMC Vet. Res..

[73] Anderson A., Hartmann K., Leutenegger C.M., Proksch A.L., Mueller R.S., Unterer S. (2017). Role of canine circovirus in dogs with acute haemorrhagic diarrhoea. Vet. Rec..

[74] Grassi L., Menandro M.L., Obber F., Drigo M., Legnardi M., Pasotto D., Tucciarone C.M., Faustini G., Citterio C., Cecchinato M. (2022). Investigation of *Carnivore protoparvovirus* 1 and *Amdoparvovirus* infections in red fox populations of the Italian Dolomites. Vet. Res. Commun..

[75] Han S.C., Guo H.C., Sun S.Q., Shu L., Wei Y.Q., Sun D.H., Cao S.Z., Peng G.N., Liu X.T. (2015). Full-length genomic characterizations of two canine parvoviruses prevalent in Northwest China. Arch. Microbiol..

[76] Battilani M., Balboni A., Ustulin M., Giunti M., Scagliarini A., Prosperi S. (2011). Genetic complexity and multiple infections with more Parvovirus species in naturally infected cats. Vet. Res..

[77] Domingo E. (1997). Rapid evolution of viral RNA genomes. J. Nutr..

[78] Truyen U., Müller T., Heidrich R., Tackmann K., Carmichael L.E. (1998). Survey on viral pathogens in wild red foxes (*Vulpes vulpes*) in Germany with emphasis on parvoviruses and analysis of a DNA sequence from a red fox parvovirus. Epidemiol. Infect..

[79] Allison A.B., Harbison C.E., Pagan I., Stucker K.M., Kaelber J.T., Brown J.D., Ruder M.G., Keel M.K., Dubovi E.J., Holmes E.C. (2012). Role of multiple hosts in the cross-species transmission and emergence of a pandemic parvovirus. J. Virol..

[80] Franzo G., Menandro M.L., Grassi L. (2021). Canine circovirus in foxes from northern Italy: Where did it all begin?. Pathogens.

[81] Ndiana L.A., Lanave G., Vasinioti V., Desario C., Martino C., Colaianni M.L., Pellegrini F., Camarda A., Berjaoui S., Sgroi G. (2022). Detection and genetic characterization of canine adenoviruses, circoviruses, and novel cycloviruses from wild carnivores in Italy. Front. Vet. Sci..

[82] Schuller S., Francey T., Hartmann K., Hugonnard M., Kohn B., Nally J.E., Sykes J. (2015). European consensus statement on leptospirosis in dogs and cats. J. Small Anim. Pract..

[83] Clark L.G., Kresse J.I., Marshak R.R., Hollister C.J. (1960). *Leptospira pomona* infection in an Eastern red fox (*Vulpes fulva fulva*). Nature.

[84] Alić A., Šupić J., Goletić T., Rešidbegović E., Lutvikadić I., Hodžić A. (2021). A unique case of fatal coinfection caused by *Leptospira* spp. and *Hepatozoon* canis in a red fox cub (*Vulpes vulpes*). Pathogens.

[85] Huber D., Habuš J., Turk N., Vinicki K., Šoštarić-Zuckermann I.C. (2023). Acute lethal leptospirosis in a red fox (*Vulpes vulpes*). J. Comp. Pathol..

[86] Müller H., Winkler P. (1994). Ergebnisse serologischer untersuchungen auf *Leptospira*-antikörper bei Füchsen [Results of serological studies of *Leptospira* antibodies in foxes]. Berl. Münch. Tierärztl. Wochenschr..

[87] Milas Z., Turk N., Janicki Z., Slavica A., Starešina V., Barbić L.J., Lojkić M., Modrić Z. (2006). Leptospiral antibodies in red foxes (*Vulpes vulpes*) in northwest Croatia. Vet. Arhiv..

[88] Millán J., Candela M.G., López-Bao J.V., Pereira M., Jiménez M.A., León-Vizcaíno L. (2009). Leptospirosis in wild and domestic carnivores in natural areas in Andalusia, Spain. Vector-Borne Zoonotic Dis..

[89] Slavica A., Dezdek D., Konjevic D., Cvetnic Z., Sindicic M., Stanin D., Habus J., Turk N. (2011). Prevalence of leptospiral antibodies in the red fox (*Vulpes vulpes*) population of Croatia. Vet. Med..

[90] Tagliabue S., Figarolli B.M., D’Incau M., Foschi G., Gennero M.S., Giordani R., Natale A., Papa P., Ponti N., Scaltrito D. (2016). Serological surveillance of Leptospirosis in Italy: Two-year national data (2010–2011). Vet. Ital..

[91] Żmudzki J., Arent Z., Jabłoński A., Nowak A., Zębek S., Stolarek A., Bocian Ł., Brzana A., Pejsak Z. (2018). Seroprevalence of 12 serovars of pathogenic *Leptospira* in red foxes (*Vulpes vulpes*) in Poland. Acta Vet. Scand..

[92] Žele-Vengušt D., Lindtner-Knific R., Mlakar-Hrženjak N., Jerina K., Vengušt G. (2021). Exposure of free-ranging wild animals to zoonotic *Leptospira interrogans* sensu stricto in Slovenia. Animals.

[93] Kuhnert P., Brodard I., Ackermann S., Schierack P., Jores J. (2023). Serological and molecular detection as well as typing of *Leptospira* spp. in foxes, raccoons, and other wild carnivores in North-Eastern Germany, 2021–2022. Heliyon.

[94] Straub M.H., Foley J.E. (2020). Cross-sectional evaluation of multiple epidemiological cycles of *Leptospira* species in peri-urban wildlife in California. J. Am. Vet. Med. Assoc..

[95] Piredda I., Scarpa F., Sanna D., Casu M., Ponti M.N., Chisu V. (2021). Draft genome sequences of four different strains belonging to *Leptospira interrogans* serovar Pomona isolated from mammals in the island of Sardinia, Italy. Microbiol. Resour. Announc..

[96] Ebani V.V., Trebino C., Guardone L., Bertelloni F., Cagnoli G., Nardoni S., Sel E., Wilde E., Poli A., Mancianti F. (2022). Occurrence of bacterial and protozoan pathogens in red foxes (*Vulpes vulpes*) in central Italy. Animals.

[97] Helman S.K., Tokuyama A.F.N., Mummah R.O., Stone N.E., Gamble M.W., Snedden C.E., Borremans B., Gomez A.C.R., Cox C., Nussbaum J. (2023). Pathogenic *Leptospira* are widespread in the urban wildlife of southern California. Sci. Rep..

[98] Boniotti M.B., Gelmini L., Carra E., Figarolli B.M., D’incau M., Tagliabue S. *Leptospira Interrogans* serogroup Australis in hedgehog in Northern Italy. Proceedings of the International Leptospirosis Society of the Conference.

[99] Trogu T., Canziani S., Salvato S., Bianchi A., Bertoletti I., Gibelli L.R., Alborali G.L., Barbieri I., Gaffuri A., Sala G. (2021). Canine distemper outbreaks in wild carnivores in Northern Italy. Viruses.

[100] Ricci I., Cersini A., Manna G., Marcario G.A., Conti R., Brocherel G., Grifoni G., Eleni C., Scicluna M.T. (2021). A canine distemper virus retrospective study conducted from 2011 to 2019 in Central Italy (Latium and Tuscany regions). Viruses.

[101] Trogu T., Castelli A., Canziani S., Tolini C., Carrera M., Sozzi E., Lelli D., Tosi G., Fiorentini L., Di Donato A. (2022). Detection and molecular characterization of canine distemper virus in wildlife from Northern Italy. Pathogens.

