# Supplementary material for: Molecular Detection of Viral and Bacterial Pathogens in Red Foxes (Vulpes vulpes) from Italy
Source: Animals (Basel). 2024 Jul 3;14(13):1969. doi: 10.3390/ani14131969 (PMC11240561; doi:10.3390/ani14131969)
Supplement: Supplementary file 1 [file animals-14-01969-s001.zip › animals-2995021-supplementary.pdf]

Supplementary Material and Methods. Details on *Protoparvovirus carnivoran* 1, *Canine mastadenovirus*, *Circovirus canine*, *Canine distemper virus* and *Leptospira* spp. nucleic acids detection and nucleotide sequencing for genetic characterization.

The detection of the nucleic acid of the pathogens investigated was carried out from different tissue samples: *Protoparvovirus carnivoran* 1 (PPVC-1) and *Circovirus canine* (CanineCV) DNA from spleen, liver and intestine samples, *Canine mastadenovirus* (CAdV) DNA from spleen, liver, intestine and kidney samples, *Canine distemper virus* (CDV) RNA from spleen or liver samples and *Leptospira* spp. DNA from kidney samples.

DNA extraction from all the organs sampled was performed using the NucleoSpin Tissue kit (Macherey-Nagel, Düren, Germany) according to the manufacturer's instructions. The extracted DNA was eluted in 100 µL of elution buffer and stored at -20°C until analysis. RNA extraction from spleen and liver samples was performed using the RNeasy kit (Qiagen, Hilden, Germany) according to the manufacturer's instructions. The extracted RNA was eluted in 40 µL of RNase-free water and stored at -80°C until analysis.

The detection of PPVC-1, CAdV, CanineCV and *Leptospira* spp. DNA was carried out with four specific SYBR Green real-time PCR (qPCR) assays, each of which performed using the PowerUp SYBR Green Master Mix (Thermo Fisher Scientific, Life Technologies, Carlsbad, CA, USA), following the manufacturer's instructions. The detection of CDV RNA was carried out with a specific SYBR Green reverse transcriptase qPCR (RT-qPCR) assay, performed using the Power SYBR Green RNA-to-CT 1-Step kit (Life Technologies, California, USA) following the manufacturer's instructions. Each reaction was carried out in a total volume of 20 µL and using the StepOnePlus Real-Time PCR System (Thermo Fisher Scientific, Life Technologies, Carlsbad, CA, USA).

The presence of FPV and CPV-2 DNA was investigated by using a qPCR targeting a fragment of 99 nucleotides (nts) in the main capsid protein VP2 gene, with the primers CPV-qPCR_For (5'-

AGC TAC TAT TAT GAG ACC AGC TGA G -3') and CPV-qPCR_Rev (5'- CCT GCT GCA ATA GGT GTT TTA A -3'). The presence of CAdV DNA was investigated by using a qPCR targeting a fragment of 166 nts in the E3 and U-exon genes, with the primers CAdV-qPCR_For3 (5'- CTG ASA CTG CWA TRM CTA TAT AYA TTT CCA -3') and CAdV-qPCR_Rev2 (5'- GAC ATA GAR ACT CAG GAC CCA GA -3'), and able to discriminate the two viral types (CAdV-1 and CAdV-2) on the basis of melting curve analysis. The presence of CanineCV DNA was investigated by using a qPCR targeting a fragment of 132 nts in the intergenic region (IR) between the ends of the two major open reading frames (ORFs), with the primers Circo-909-932-qPCR-For (5'- CTG AAA GAT AAA GGC CTC TCG CT -3') and Circo-1020-1040-qPCR-Rev (5'- AGG GGG GTG AAC AGG TAA ACG -3'). The presence of *Leptospira* spp. DNA was investigated by using a qPCR targeting a fragment of 242 nts in the Lipl32 gene, with the primers LipL32-45F (5'- AAG CAT TAC CGC TTG TGG TG -3') and LipL32-286R (5'- GAA CTC CCA TTT CAG CGA TT -3'). The thermal cycling of all reactions consisted of initial DNA denaturation at 95°C for 5 min followed by 45 cycles at 95°C for 15 s and 60°C for 1 min.
The presence of CDV RNA was investigated by using a RT-qPCR targeting a fragment of 114 nts in the P gene, with the primers qCDVF4 (5'- GTC GGT AAT CGA GGA TTC GAG AG -3') and qCDVR3 (5'- GCC GAA AGA ATA TCC CCA GTT AG -3'). The thermal cycling of the reaction consisted in reverse transcription at 48°C for 30 min, followed by the activation of AmpliTaq Gold DNA Polymerase at 95°C for 10 min and then 40 cycles at 95°C for 15 s and 60°C for 1 min.
The melting experiment for the evaluation of the specificity of each reaction was performed after the last extension step by a continuous increment from 55°C to 98°C and specific melting temperature were about 77°C for FPV and CPV-2, 73°C for CAdV-1, 80°C for CAdV-2, 93°C for CanineCV, 78°C for CDV and 82°C for Leptospira spp. Viral nucleic acid copy number determination was carried out by absolute quantification using the standard curve method. Serial 10-fold dilutions of a plasmid (pCR4 plasmid, TOPO TA Cloning Kit, Life Technologies, USA) containing one copy of the respective viral target sequence were used as external standards for the

construction of the assay standard curve by plotting the plasmid copy number against the corresponding threshold cycle values. The limit of detection (LOD) of the reactions were determined based on the highest dilution of recombinant plasmid possible to amplify with good reproducibility and was found to be one copy/µlL for FPV, CPV-2, CAdV-1, CAdV-2, CDV and Leptospira spp, and five copies/µlL for CanineCV. The DNA or RNA samples and standards were repeated within each run in duplicate. A no template control, consisting of ultrapure water, and a negative extraction control underwent analysis simultaneously. Samples showing an exponential increase in the fluorescence curve, a target amount greater than or equal to the LOD and a specific melting peak in both replicates were considered positive.

The viruses identified with the screening qPCRs were genetically characterised by the integrated use of amplification assays, sequencing and bioinformatics analysis. For red foxes that showed specific qPCR products for the same pathogen in more than one organ, the biological matrix showing the highest amount of target DNA or no non-specific products was subjected to subsequent analyses.

Each end-point PCR assay was performed using the Phusion Hot Start II DNA Polymerase (Thermo Fisher Scientific, Life Technologies, Carlsbad, CA, USA), containing a high-fidelity DNA polymerase, according to the manufacturer's instruction, in a total volume of 50 µlL containing 20 µM of each primer, 5X HF buffer, 2.5 mM dNTP, 2 U/µlL Phusion Hot Start II DNA Polymerase and 5 µlL of DNA extract. A positive control, consisting of a sure positive sample, and a no template control underwent analysis simultaneously.

For the identified PPVC-1, a fragment of the VP2 gene was amplified using a hemi-nested PCR with the primers P3 (5′- CCA TTT CTA AAT TCT TTG -3′), VPR (5'- TTT CTA GGT GCT AGT TGA G -3') and P4 (5′- AAG TCA GTA TCA AAT TCT T -3′). The canine parvovirus 2b (CPV-2b) 190/2018 (MW829216) was used as positive control.

For the identified CAdV, the complete hexon and fiber genes were amplified using two end-point PCR with the primers CAdV-Hexon-For1 (5′- GAA GTT TGC CGA CCC TGT C -3′) and CAdV-

Hexon-Rev1 (5′- ACT ATG GCT CGC AGC TCT TC -3′), and CAdV-Fiber-For1 (5′- ATG TGG TCT CTC CCR ACA GC -3′) and CAdV-Fiber-Rev1 (5′- ACT TTT CCT GAA GGC GGY AG -3′), respectively. The CAdV-1 452/2017 (MW829199 and MW829200) was used as positive control.

For the identified CanineCV, the complete viral genome was amplified integrating rolling circle amplification (RCA) and PCR methods. The RCA was performed on the positive samples to increase the amount of circular DNA using the TempliPhi Amplification kit (GE Healthcare, Chicago, IL, USA) following the manufacturer's instructions. Subsequently, viral DNA was amplified by two PCR with the primers CaCV 1020-1040 For (5′- CGT TTA CCT GTT CAC CCC CCT -3′) and CaCV 909-931 Rev (5′- AGC GAG AGG CCT TTA TCT TTC AG -3′), and CaCV 3'-3' For (5′- ATG GTG GGA TGG CTA CGA TG -3′) and CaCV 3'-3' Rev (5′- CAA GGA AGA GGG AAT GCT ACA AG -3′), respectively. The CanineCV 449/2017 (MW829203) was used as positive control.

Five microliters of each amplicon were separated by electrophoresis in a 1-2 % (W/V) agarose gel stained with Midori Green Advance DNA Stain (Nippon Genetics, Chiyodaku, Tokyo, Japan) in 1 X Tris-acetate ethylene diamine tetra-acetic acid (TAE) buffer, together with a GeneRuler 100 nts or 1000 nts DNA Ladder (Thermo Fisher Scientific, USA), and visualised with ultraviolet (UV) light. Amplicons of the expected size were considered positive, purified using the QIAquick PCR Purification Kit (Qiagen, Hilden, Germany) according to the manufacturer's instructions and sequenced with forward and reverse primers by Sanger method (BioFab Research, Rome, Italy).

Supplementary Table S1. Red foxes tested positive for at least one pathogen and gross findings.

Red foxes	PPVC-1			CAdV				CanineCV			CDV		*Leptospira* spp.	Main gross findings
	Intestine	Spleen	Liver	Intestine	Spleen	Liver	Kidney	Intestine	Spleen	Liver	Spleen	Liver	Kidney	
154/2022	neg	POS S<br>37.2<br>$1.4x10^{1}$<br>CPV-2b	NA	neg	neg	NA	neg	POS S<br>17<br>$4.4x10^{6}$<br>CanineCV	POS<br>15.1<br>$1.3x10^{7}$	NA	neg	NA	neg	Culling
155/2022	neg	neg	NA	neg	neg	NA	neg	POS<br>29.6<br>$3.1x10^{3}$	POS S<br>22<br>$2.5x10^{5}$<br>CanineCV	NA	neg	NA	neg	Culling
156/2022	neg	POS S<br>39.1<br>$3x10^{0}$<br>CPV-2b	NA	neg	neg	NA	neg	POS<br>34.6<br>$1.7x10^{2}$	POS S<br>17.5<br>$3.2x10^{6}$<br>CanineCV	NA	neg	NA	neg	Culling
157/2022	neg	neg	NA	neg	neg	NA	neg	neg	POS<br>37<br>$4.4x10^{1}$	NA	neg	NA	neg	Culling
158/2022	neg	neg	NA	neg	neg	NA	neg	POS<br>37.4<br>$3.5x10^{1}$	neg	NA	neg	NA	neg	Culling
246/2022	neg	neg	NA	neg	neg	NA	neg	POS<br>33.2<br>$3.9x10^{2}$	POS S<br>20<br>$8.2x10^{5}$<br>CanineCV	NA	neg	NA	neg	Culling
250/2022	neg	POS<br>38.1<br>$5.6x10^{0}$	NA	neg	neg	NA	NA	neg	neg	NA	neg	NA	NA	Trauma
251/2022	neg	POS<br>36.2<br>$1.9x10^{1}$	NA	neg	neg	NA	NA	neg	neg	NA	neg	NA	NA	Trauma
827/2022	POS<br>36.3<br>$8.5x10^{0}$	POS S<br>32.6<br>$9x10^{1}$<br>CPV-2b	NA	neg	neg	NA	neg	neg	neg	NA	neg	NA	neg	Trauma
828/2022	POS S<br>36.1<br>$9.8x10^{0}$<br>CPV-2b	neg	NA	neg	neg	NA	neg	neg	neg	NA	neg	NA	neg	Culling
829/2022	POS S C<br>32<br>$1.3x10^{2}$<br>CPV-2b + FPV	POS<br>36.4<br>$8x10^{0}$	NA	neg	neg	NA	neg	neg	neg	NA	neg	NA	neg	Trauma
830/2022	POS<br>32.5<br>$9.8x10^{1}$	POS S<br>31.7<br>$1.6x10^{2}$<br>CPV-2a	NA	neg	neg	NA	NA	neg	neg	NA	neg	NA	NA	Trauma

1422/2022	POS S<br>33.6<br>1.9x10^{2}<br>FPV	neg	NA	POS<br>37.9<br>4.4x10^{1}<br>CAdV-2	neg	NA	neg	POS S<br>24.2<br>6.7x10^{4}<br>CanineCV	POS<br>31.2<br>1x10^{3}	NA	neg	NA	neg	Trauma
1425/2022	neg	POS<br>36.9<br>2.8x10^{1}	NA	neg	neg	NA	NA	neg	neg	NA	neg	NA	NA	Trauma
1433/2022	POS<br>38.3<br>6.2x10^{0}	neg	NA	neg	neg	NA	neg	neg	neg	NA	neg	NA	neg	Culling
1435/2022	POS<br>38.5<br>5.3x10^{0}	neg	NA	neg	neg	NA	neg	neg	neg	NA	neg	NA	neg	Culling
1441/2022	POS<br>37.8<br>3.2x10^{1}	neg	NA	neg	neg	NA	neg	neg	neg	NA	neg	NA	neg	Culling
1784/2022	neg	neg	NA	neg	POS<br>34.5<br>5.2x10^{2}<br>CAdV-1	NA	neg	neg	neg	NA	neg	NA	neg	Culling
1794/2022	neg	neg	NA	neg	neg	NA	neg	POS<br>39.2<br>2.1x10^{1}	POS<br>40.7<br>9.3x10^{0}	NA	neg	NA	neg	Culling
1795/2022	neg	neg	NA	neg	neg	NA	neg	neg	POS S<br>17.8<br>4x10^{6}<br>CanineCV	NA	neg	NA	neg	Culling
1798/2022	neg	neg	NA	neg	POS S<br>36.9<br>1.2x10^{2}<br>CAdV-1	NA	neg	neg	neg	NA	neg	NA	neg	Gastrointestinal signs
1805/2022	neg	neg	NA	neg	neg	NA	neg	neg	neg	NA	neg	NA	POS S<br>39.6<br>2.4x10^{1}<br>*L. interrogans* ST198	Culling
1806/2022	neg	neg	NA	neg	neg	NA	neg	neg	neg	NA	neg	NA	POS S<br>34<br>8.9x10^{1}<br>*L. interrogans* ST198	Culling
463/2023	neg	neg	NA	neg	neg	NA	neg	POS<br>36.9<br>6.2x10^{1}	POS<br>33.3<br>3.1x10^{2}	NA	neg	NA	neg	Culling
464/2023	neg	neg	NA	neg	neg	NA	neg	POS<br>34.1<br>2x10^{2}	POS S<br>17.8<br>1.8x10^{6}<br>CanineCV	NA	neg	NA	neg	Culling
465/2023	neg	neg	NA	neg	neg	NA	neg	POS<br>31.9<br>7x10^{2}	POS S<br>22.9<br>1x10^{5}<br>CanineCV	NA	neg	NA	neg	Culling

468/2023	POS S<br>37.4<br>7.8x10^{0}<br>FPV	neg	NA	neg	neg	NA	neg	neg	neg	NA	neg	NA	neg	Trauma
469/2023	POS S<br>39<br>1.9x10^{0}<br>FPV	neg	NA	neg	neg	NA	neg	neg	neg	NA	neg	NA	neg	Trauma
471/2023	neg	neg	NA	neg	neg	NA	neg	neg	POS<br>34.7<br>1.5x10^{2}	NA	neg	NA	neg	Culling
472/2023	neg	neg	NA	neg	neg	NA	neg	POS<br>28.2<br>5.4x10^{3}	POS S<br>14.8<br>9.3x10^{6}<br>CanineCV	NA	neg	NA	neg	Culling
473/2023	neg	neg	NA	neg	neg	NA	neg	POS<br>38.1<br>2.3x10^{1}	POS S<br>37<br>4.1x10^{1}<br>CanineCV	NA	neg	NA	neg	Trauma
474/2023	neg	neg	NA	neg	neg	NA	neg	POS<br>38.1<br>2.3x10^{1}	POS S<br>35.4<br>1x10^{2}<br>CanineCV	NA	neg	NA	neg	Culling
475/2023	neg	neg	NA	neg	neg	NA	neg	POS<br>40.4<br>6.4x10^{0}	POS<br>38.6<br>1.7x10^{1}	NA	neg	NA	neg	Trauma
480/2023	POS<br>37.5<br>9.7x10^{0}	POS<br>37.2<br>1.3x10^{1}	NA	neg	neg	NA	neg	neg	neg	NA	neg	NA	neg	Trauma
481/2023	POS S<br>31.4<br>4.1x10^{2}<br>FPV	POS<br>33.3<br>1.1x10^{2}	NA	neg	neg	NA	neg	neg	neg	NA	neg	NA	neg	Pneumonia
483/2023	neg	NA	POS<br>38<br>5.1x10^{0}	neg	NA	neg	neg	neg	NA	neg	NA	neg	neg	Mange
485/2023	neg	neg	NA	neg	neg	NA	neg	POS<br>37.6<br>1.2x10^{2}	POS S<br>21.2<br>5.1x10^{5}<br>CanineCV	NA	neg	NA	neg	Culling
489/2023	POS S<br>36.7<br>1.6x10^{1}<br>CPV-2b	neg	NA	neg	neg	NA	neg	neg	POS S<br>35.5<br>2.2x10^{2}<br>CanineCV	NA	neg	NA	neg	Culling
492/2023	neg	POS<br>39<br>3.2x10^{0}	NA	neg	neg	NA	neg	POS<br>36.8<br>1.1x10^{2}	POS<br>34.8<br>3.2x10^{2}	NA	neg	NA	neg	Culling

In positive samples the following data are reported: mean threshold cycle (Ct); mean quantity expressed in copies/µl; and, when sequenced, the virus identified.

PPVC-1: Protoparvovirus carnivoran 1; CAdV: Canine mastadenovirus; CanineCV: Circovirus canine; CDV: Canine distemper virus; CPV-2: Canine parvovirus type 2; FPV: Feline panleukopenia virus; POS: positive; neg: negative; S: sequencing; C: cloning; NA: not available.
